# Electrode Impedance Subcomponent Analysis in Cochlear Implant Patients with Rising or Fluctuating Electrode Impedances

**DOI:** 10.3390/audiolres15020041

**Published:** 2025-04-12

**Authors:** Aniket A. Saoji, Madison K. Graham, Melissa D. DeJong, Joscelyn R. K. Martin, Joerg Pesch, Filiep J. Vanpoucke

**Affiliations:** 1 Department of Otolaryngology-Head and Neck Surgery, Mayo Clinic, Rochester, MN 55905, USA; graham.madison@mayo.edu (M.K.G.); dejong.melissa@mayo.edu (M.D.D.); martin.joscelyn@mayo.edu (J.R.K.M.); 2 Cochlear Ltd., Advanced Innovation, 2800 Mechelen, Belgium; jpesch@cochlear.com (J.P.); fvanpoucke@cochlear.com (F.J.V.)

**Keywords:** cochlear implants, electrode impedances, transimpedance matrix

## Abstract

Background/Objectives: Electrode impedance is crucial for optimizing cochlear implant (CI) stimulation and hearing outcomes. While typically stable, some patients experience unexplained impedance fluctuations. This study used electrode impedance subcomponent analysis to identify the subcomponents contributing to these impedance fluctuations. Methods: This study analyzed clinical electrode impedances and transimpedance matrix (TIM) measurements in 10 CI patients with Nucleus devices (CI422, CI522, or CI622 electrode arrays) who exhibited fluctuating or rising electrode impedances. TIM measurements used a cathodic-leading biphasic pulse (110 CLs, 75 µs/phase, 7 µs interphase interval). Electrode impedances were determined at 6, 12, 18, 24, and 75 µs, and subcomponents (access resistance [near-field/far-field] and polarization impedance [Warburg capacitance/Faraday resistance]) were calculated. Results: Both access resistance and polarization impedance changes contributed to impedance fluctuations. Large changes in near-field resistance compared to far-field resistance were associated with increased resistance to current flow closer to the surface of the electrode. The decreased double-layer capacitance and slightly increased Faraday resistance further suggested increased resistance to charge transfer at the electrode–electrolyte interface. Conclusions: Electrode impedance subcomponent analysis reveals changes in the electrochemical reaction at the electrode surface that cause fluctuating or rising CI electrode impedances.

## 1. Introduction

In cochlear implants (CIs), electrode impedance measurement plays a role in optimizing power requirements, establishing electrode integrity, and determining electrical stimulation parameters such as the pulse duration, pulse amplitude, and stimulation rate. During the measurement of electrode impedances, a biphasic pulse with a current (I) is delivered to the stimulating electrode, and the voltage (V) generated is measured. The electrode impedance is calculated by dividing the measured voltage V by the current amplitude I. The electrode impedance derived from the voltage measured at the end of the leading phase of the biphasic pulse is known to reflect the total electrode impedance, which is a combination of the tissue impedance and electrode contact impedance. The increase in stimulation voltage measured at the onset of the biphasic pulse is known as the access voltage (V_a_). The difference between the total voltage (V_T_) measured at the end of the first phase of the biphasic pulse and the access voltage is known as the polarization voltage (V_p_). The electrode impedances derived from these two voltage (V_a_ and V_p_) measurements are known as access resistance (R_a_) and polarization impedance (Z_p_) [1,2,3,4,5,6,7].

In monopolar mode, R_a_ represents the resistance to current flow between the intracochlear electrode and the extracochlear ground or return electrode. R_a_ is the sum of near-field resistance (R_n_) and far-field resistance (R_f_) connected in series. R_n_ represents the resistance to current flow through the wire connecting the implant to the electrode, the platinum electrode, the electrode–electrolyte interface, and the very close vicinity of the electrode contact. R_n_ can monitor localized changes on the electrode surface and the electrode–electrolyte interface. R_f_ represents the resistance to current flow through the cochlear structures and surrounding extracochlear tissue. Polarization impedance (Z_p_) is modellable by a simplified electrical circuit consisting of Warburg impedance (C_p_) and Faradaic resistance (R_p_). C_p_ reflects the double-layer capacitance formed by the accumulation of positive ions in the perilymph against the negatively charged platinum electrode or vice versa. This process is reversible by involving the non-Faradaic charge injection without electron transfer. R_p_ models the charge transfer reactions that become active upon the sufficient charging of the double layer. These reactions involve electron exchange between the platinum electrode and the perilymph electrolytes. Electrode impedance subcomponent analysis can be defined using the following formulas:

Total impedance (Z_T_) = access resistance (R_a_) + polarization impedance (Z_p_)

R_a_ = near-field (R_n_) + far-field (R_f_) resistance

Z_p_ = Warburg capacitance (C_p_) and Faradaic resistance (R_p_)

During programming visits, repeated clinical electrode impedance measurements show high re-test reliability with a median difference of 123 Ω [8,9]. Choi et al. suggested that a median increase in electrode impedance ≥4 kΩ should be considered significant and may reflect changes in the protein composition and cell contents of the inner-ear fluids [9]. Clinical electrode impedances have been shown to vary with the inflammation of the inner ear [9,10], presence of middle-ear fluid [11], loss of residual hearing [12,13], and introduction of new medical conditions or medications. Neuburger et al. attributed changes in clinical electrode impedances to exceeding voltage compliance with electrical stimulation delivery [14]. Both steroid prescriptions and modifications to electrical stimulation parameters (i.e., widening pulse duration) have shown impedance stabilization [14,15].

A small subset of patients exhibits persistent variations in clinical electrode impedances following treatment, even in the absence of identifiable or well-understood underlying causes. In two CI patients with fluctuating electrode impedances, Saoji et al. found that electrical stimulation increased electrode impedance, and device rest decreased it [16]. The mechanism behind this increase in impedance after electrical stimulation remains unclear. We observed that rising electrode impedances are not always persistent; an electrode might show increased impedance one day and be stable the next day. This impedance rise is localized and electrode-specific, triggered by electrical stimulation on individual electrodes, without affecting neighboring ones. Notably, disabled electrodes do not exhibit this increase. Patients with this fluctuating impedance pattern often report significant volume variations and poor sound quality, leading to increased clinic visits. To manage these issues, we have implemented device rest as a strategy. This includes periods of complete device rest or the use of alternating programs where either even or odd electrodes are disabled. By cycling between these programs, we provide targeted rest to specific electrodes, which has demonstrated improvements in sound quality for these patients.

The mechanism behind fluctuating or rising electrode impedances is not fully understood. However, impedance subcomponent analysis offers a valuable tool to investigate these fluctuations by distinguishing between near- and far-field changes within the cochlea. The ability to lower impedance through device rest offers a valuable opportunity to investigate total electrode impedance and its components in these patients.

This exploratory study utilized electrode impedance subcomponent analysis in 10 Nucleus CI recipients exhibiting unexplained electrode impedance fluctuations without apparent middle- or inner-ear pathology. The aim was to determine if electrode impedance subcomponent analysis could identify specific impedance subcomponents associated with these fluctuations.

## 2. Methods

A retrospective analysis of clinical electrode impedances and transimpedance matrix measurements performed during routine clinical visits is reported for 10 Nucleus CI patients with fluctuating or rising electrode impedances. Approximately 5% of our patient population experiences persistent electrode impedance fluctuations. While transient fluctuations are common, typically over time, this subset of patients demonstrated fluctuations throughout the duration of implant use. Table 1 shows the subject demographics, the device type, implant duration, disabled electrodes, and the timing of clinical visits (from the day of implantation) for our study population. Impedance fluctuations were observed in all subjects from their first follow-up visit post-activation. All subjects reported here had the lateral wall electrode array, which is the most preferred electrode type used at our CI center. The study protocol was approved by the Mayo Clinic Institutional Review Board (# 24-010370).

The TransImpedance Matrix (TIM) feature in Custom Sound EP 6.0 software from Cochlear Ltd. (Sydney, Australia) was used to perform these measurements. Electrode voltages were measured at 6, 12, 18, 24, and 75 µs during the cathodic phase of a biphasic pulse (128 µA; 75 µs/phase; 7 µs interphase interval). A biphasic pulse was repeated to measure electrode voltage at each time point throughout the duration of the cathodic phase. The extracochlear case or MP2 electrode was used as the stimulation and recording ground electrode.

For further analysis, the impedance waveforms are described in terms of an equivalent discrete electrical circuit model [1] consisting of a resistor (R_a_, access resistance) in series with a parallel circuit consisting of a capacitor (C_p_, Warburg capacitance) and a resistor (R_p_, Faradaic resistance). The three parameters are extracted from the voltage/impedance waveform by non-linear curve fitting implemented in Matlab (Mathworks, Natick, MA, USA). When recording from a stimulating contact (impedance), the R_a_ + C_p_//R_p_ circuit model was used. Such a circuit will respond to a biphasic current pulse with a voltage response that is the sum of a voltage pulse (Ra) and a decaying exponential (C_p_//R_p_).

$$z(t)=\frac{v(t)}{I}=R_{a}+R_{p}\cdot (1-e^{-t/R_{p}\cdot C_{p}})$$

This total monopolar impedance is time-dependent. The three parameters have a graphical meaning. R_a_ corresponds to the initial impedance (t = 0 µs). The R_p_ parameter corresponds to the increase in impedance (polarization) if the pulse width is infinitely long (t = ∞ µs) In reality, this maximum polarization will never be obtained because of charge irreversibly leaking away from the contact. A constant phase circuit element (CPE) is more appropriate for long pulse durations. The C_p_ parameter is inversely proportional to the initial slope of the polarization; e.g., a value C_p_ = 5-nF implies an initial increase in the total impedance of 200 Ω/µs. This template was matched against the diagonal elements of the TIM recordings. When recording from a non-stimulating contact (off-diagonal elements of the transimpedance matrix), the recording contact is not charged. Therefore, the model was simplified to only the parameter R_a_.

The access resistance for all recordings forms a 22 × 22 matrix. The diagonal elements (recording on the stimulating contact) are typically much higher than the off-diagonal elements. Two factors contribute to the peak: first, the voltage in the cochlear duct at the stimulation contact being higher than that on neighboring contacts due to passive conduction (tissue component) because of far-field resistance; second, a local resistive contribution which is part of the interface impedance (near-field R_n_). The sum of both is the access resistance. Non-linear curve fitting is used to take into account all non-diagonal recordings for each stimulating electrode [17]. For every stimulation position, a two-sided curve was fitted containing a linear and exponential term:

$$y=A+B\cdot (x-x_{0})+C\cdot {exp}^{-abs(x-x_{0})/D}$$

with different coefficient sets toward the apex and base satisfying a constraint that they share a common intersection point at the stimulation position. This intersection value is taken as the R_f_ contribution, whereas R_n_ is defined as its difference from the total R_a_ value. While this method generally works well, it carries a potential for overestimating the far-field component, particularly at the most basal and apical electrode contacts. The R_f_ values describe the electrical conduction within the cochlear duct and through the passage to the extracochlear ground electrode. They depend on the anatomy and tissue distribution of the implanted cochlea. The R_n_, C_p,_ and R_p_ values together constitute the interface impedance. These parameters depend on the geometry of the electrode contact, its surface state, and the electrical conduction properties of the immediate tissue surrounding the electrode contact.

## 3. Results

### 3.1. Clinical Impedance Measurements

**Figure 1:**
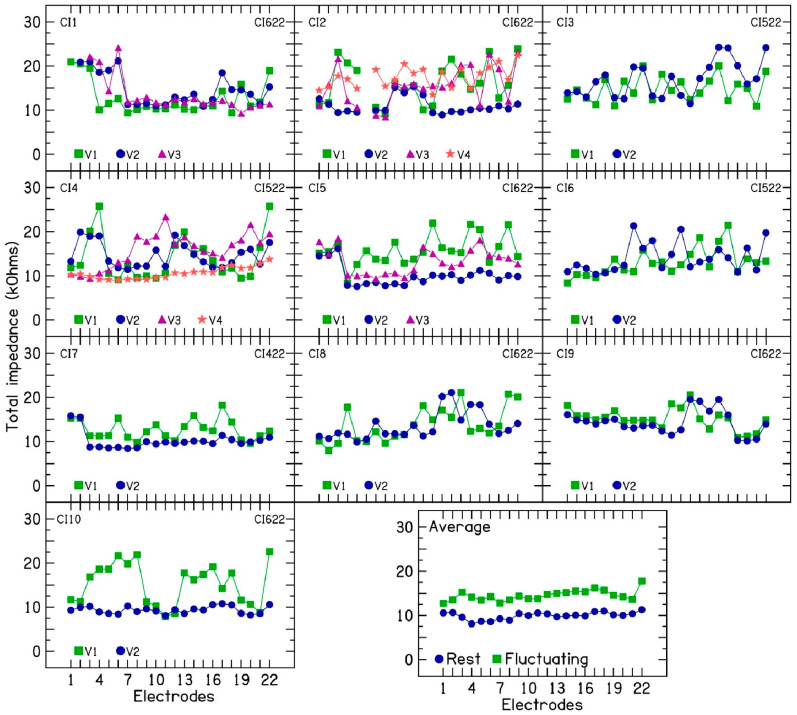
Electrode impedance measurements during different clinical visits for 10 cochlear implant patients with fluctuating electrode impedances. The abscissa shows electrodes, and the ordinate shows electrode impedance (kΩ).

The clinical impedances were recorded using a pulse duration of 25 µs and pulse amplitude of 78 CLs or approximately 80 µA. Monopolar 1 + Monopolar 2 (MP1 +2) was the return electrode. The results show large changes in electrode impedances for the 10 CI patients across clinical visits. Figure 1 shows the clinical electrode impedances measured using Custom Sound Pro 6.0 clinical programming software for the 10 CI patients. The average impedance of electrodes that decreased after device rest was also plotted, alongside the average impedance of all other electrodes that exhibited fluctuating or increasing impedance across the 10 CI patients. Across 22 electrodes in 10 CI patients, electrode impedance changes ranged from 0.02 kΩ to 16.55 kΩ, with an average of 4.73 kΩ. In five patients (CI2, CI4, CI5, CI7, and CI10), the clinical electrode impedances lowered after 2 or 3 days of device rest. Lowered impedances were plotted at visit V2 for patients CI2, CI5, CI7, and CI10. For patient CI4, lowered impedances were plotted at visit V4. Figure 1 (Average panel) displays the average difference in electrode impedance between five post-device-rest measurements (CI2, CI4, CI5, CI7, CI10) and the overall average across all other measurements for the 10 CI patients. These findings suggest an inverse relationship between electrical stimulation and electrode impedance in the patients who were prescribed device rest. Impedance values decrease during device inactivity (rest) and increase with electrical stimulation [16], which is discussed further in the Discussion Section. The remaining five patients (CI1, CI3, CI6, CI8, and CI9) also showed large fluctuations in clinical electrode impedances. Device rest was not an option for these patients due to their reliance on the implant for daily listening. Given our clinical observations of device rest’s effectiveness in managing fluctuating impedance, it is reasonable to hypothesize that these patients would have exhibited reduced electrode impedances if device rest had been prescribed.

### 3.2. Transimpedance Matrix Measurements

**Figure 2:**
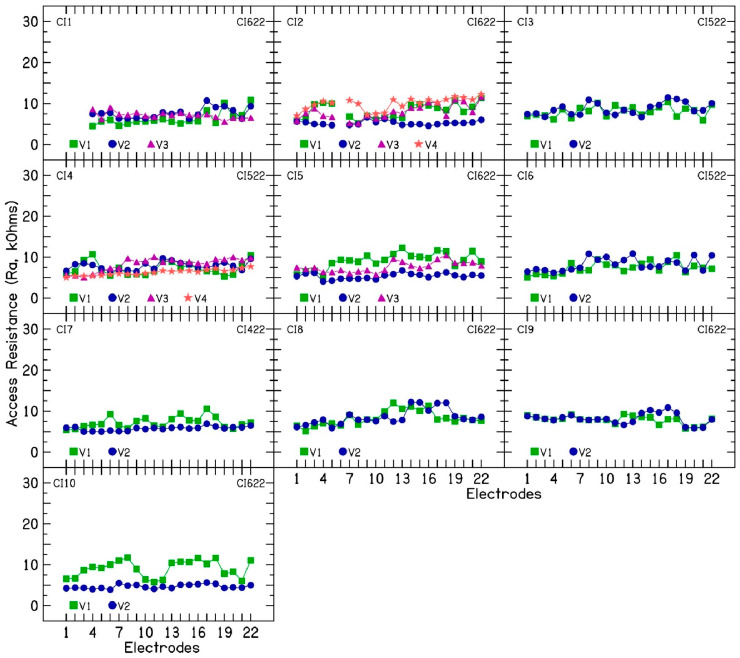
Access resistance calculated from impedance measurements performed at 6, 12, 18, 25, and 75 µs during the first phase of the biphasic pulse in 10 cochlear implant patients.

**Figure 3:**
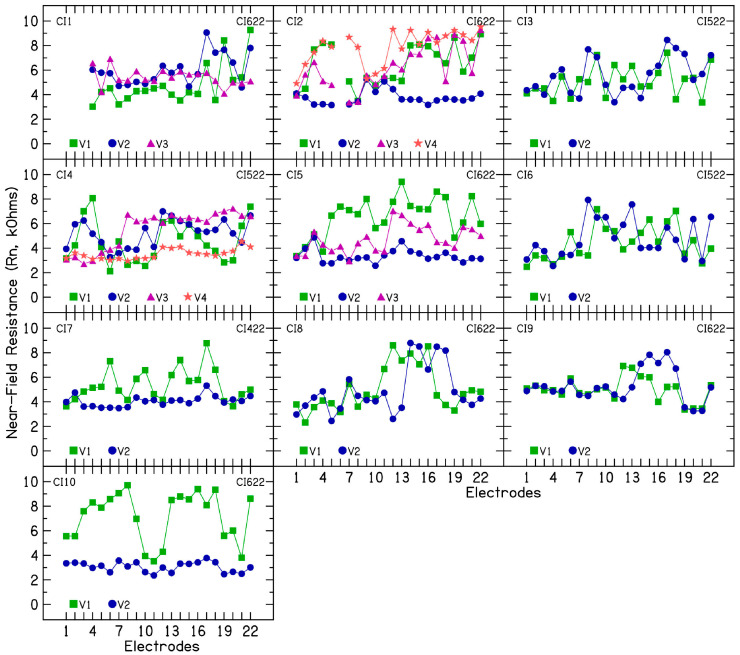
Near-field resistance in 10 cochlear implant patients with fluctuating impedances.

**Figure 4:**
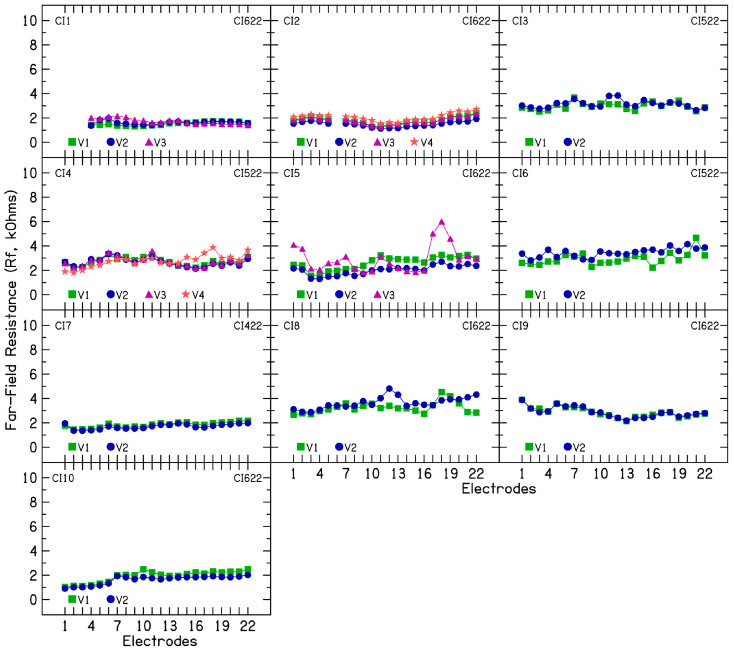
Far-field resistance in 10 cochlear implant patients with fluctuating impedances.

To investigate the nature of electrode impedance fluctuations, TIM measurements were performed during each clinical visit so that electrode impedances are broken down into constituent components. Figure 2 shows R_a_ (kΩ) as a function of the 22 intracochlear electrodes for the 10 CI patients. An average access resistance of 7.56 kΩ (SD = 1.86) was measured for the 10 CI patients across multiple clinical visits. R_a_ was broken down further into near-(Figure 3) and far-field resistance (Figure 4). An average near-field resistance of 5.13 kΩ (SD = 1.74) and far-field resistance of 2.42 kΩ (SD = 0.77) was computed for the 10 CI ears across 26 clinical visits. Visual inspection of the data and the standard deviation calculated show larger changes in near-field resistance contributing to changes in CI electrode impedances across clinical visits. These results are consistent with the notion that changes near the electrode contact area contribute to fluctuating or rising electrode impedances. The resistance of the return path to intracochlear or extracochlear ground (far-field resistance) demonstrated a minimal contribution to the observed increase in electrode impedances.

**Figure 5:**
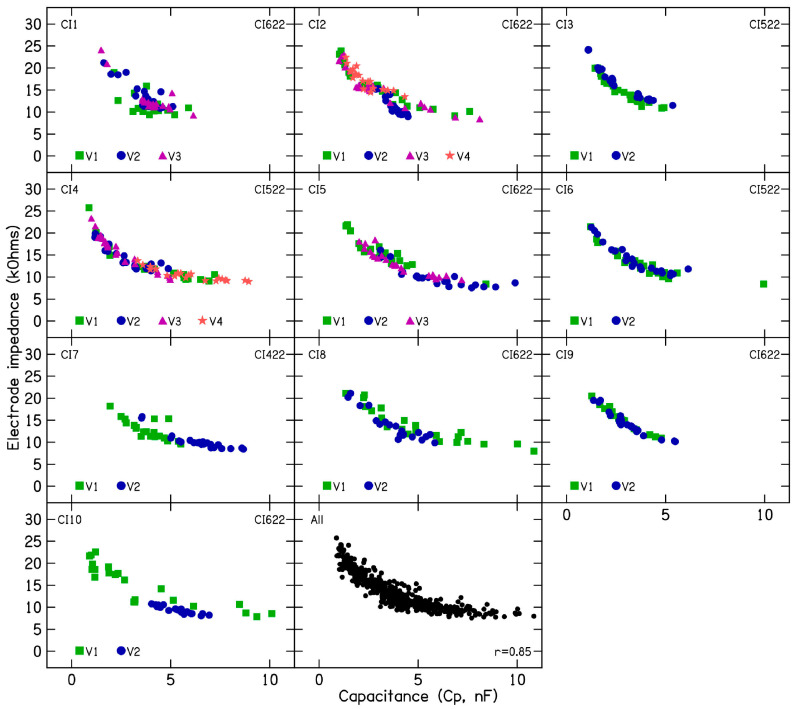
Warburg capacitance calculations in 10 CI patients with fluctuating or rising electrode impedances. The bottom middle panel includes calculations for all measured electrode impedances from these patients.

**Figure 6:**
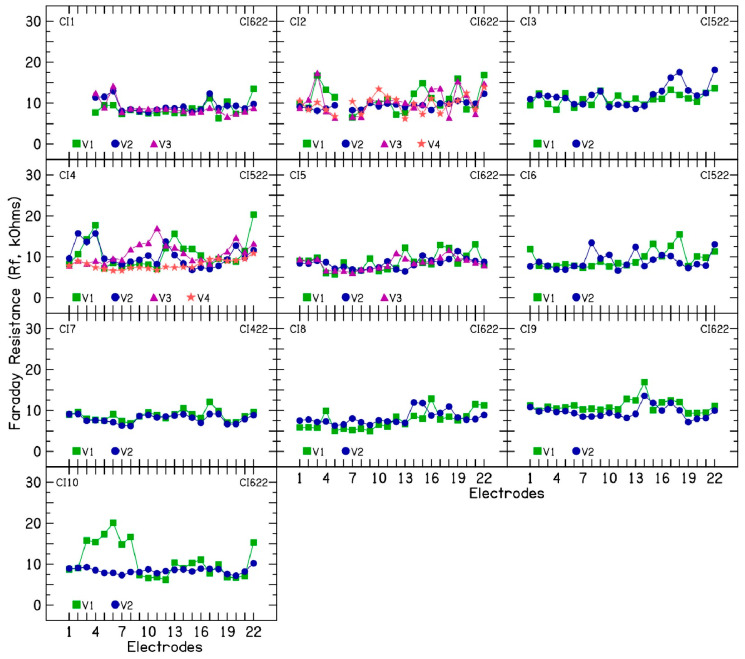
Faraday resistance calculations in patients with fluctuating impedances.

In five patients (CI2, CI4, CI5, CI7, and CI10) for whom device rest was employed to reduce electrode impedance, the average resistance (R_a_) was 5.48 kΩ (SD = 0.8), the near-field resistance was 3.55 kΩ (SD = 0.6), and the far-field resistance was 1.95 kΩ (SD = 0.57) after the rest period. Before device rest, when these patients exhibited fluctuating or increasing impedances, the average R_a_ was 8.26 kΩ (SD = 1.76), the near-field resistance was 5.64 kΩ (SD = 2.03), and the far-field resistance was 2.29 kΩ (SD = 0.67). These findings suggest a larger change in near-field resistance than in far-field resistance. Polarization impedance was calculated by subtracting the access resistance from total impedance and further divided into Warburg capacitance (Figure 5) and Faraday resistance (Figure 6). Lower capacitance numbers indicate higher polarization impedance. The results show decreasing capacitance with increasing electrode impedances and vice versa. Across 26 clinical visits in the 10 CI ears, the average capacitance was 3.83 nF (SD = 1.89). A strong correlation (r = 0.84) was found between C_p_ and total electrode impedance at 25 µs, indicating that decreasing capacitance is associated with increasing impedance. For patients CI2, CI4, CI5, CI7, and CI10, the average C_p_ was 3.27 nF (SD = 1.92), compared to 5.5 nF (SD = 1.6) after device rest resulting in lower electrode impedances.

An average Faraday resistance of 9.47 kΩ (SD = 2.4) was calculated across the 10 CI ears. Interestingly, for CIs 2, 4, 5, 7, and 10, a lower average Faraday resistance of 8.71 kΩ (SD = 1.41) was observed when electrode impedances decreased after device rest compared to 9.6 kΩ (SD = 2.84) when electrode impedances were increasing.

## 4. Discussion

This study investigated fluctuating or rising electrode impedances in 10 CI ears over multiple clinical visits. Clinical electrode impedance was measured using CI fitting software with a 25 µs cathodic-leading biphasic pulse at 78 CLs. TIM measurements were performed at 6, 12, 18, 24, and 75 µs with a 110 CL pulse. TIM data were then used to analyze impedance subcomponents, as described by [2], to understand the contribution of different subcomponents in fluctuating or rising electrode impedances. In the 10 CI patients, both access resistance and polarization impedance contributed to the observed fluctuations and increases in electrode impedance. Within access resistance, changes were more pronounced in near-field resistance than in far-field resistance. Increased near-field resistance suggests a greater resistance to current flow near the electrode surface, potentially due to alterations in the chemical reactions at the electrode–electrolyte interface [2,3]. This interpretation is supported by the decrease in double-layer capacitance, which indicates increased resistance to charge transfer at the electrode–electrolyte interface [2]. The slight increase in Faraday resistance with rising electrode impedance further supports these findings.

A counterintuitive phenomenon observed is the decrease in electrode impedance in five patients (CI2, CI4, CI5, CI7, and CI10) following a prescribed period of device rest [16]. This contrasts with the typical impedance increase seen with device rest [14]. In these patients, device rest led to decreased impedance, while subsequent electrical stimulation caused a temporary increase in some electrodes. However, the rising impedance trend resumed after stimulation restarted. The mechanism behind this unusual response is unclear and warrants further study. This phenomenon offers a unique opportunity to investigate electrode impedance subcomponents in patients with rising impedance under controlled conditions. Interestingly, disabled electrodes (e.g., electrodes 1–3 in CI5) showed no impedance changes with either device rest or the resumption of stimulation in the other active electrodes. This observation further suggests that electrical stimulation plays a key role in altering the electrochemical reactions at the electrode–electrolyte interface and that localized changes alone in the cochlear environment or the electrolyte near the surface of the electrode cannot explain the rising or fluctuating electrode impedances.

The accurate assessment of electrode impedance in CIs necessitates measuring the total electrode impedance. This measurement is typically taken at the end of the first phase in a biphasic pulse, using the same pulse duration employed for electrical stimulation on that specific electrode. In contrast, relying solely on access resistance as a proxy for electrode impedance can lead to the underestimation of the total current flow resistance within the cochlea. Access resistance is measured either at the beginning of the first phase or at the end of the second phase of the biphasic pulse. Crucially, this approach neglects the polarization component, which is known to vary depending on the pulse duration. This can result in biphasic current levels exceeding the CI compliance voltage, leading to pulse clipping, which negatively impacts sound quality and speech perception [18].

Also, measuring access resistance only can mask impedance fluctuations entirely or minimize the extent of fluctuations estimated by a clinician. Generally, electrode impedances are assumed to fluctuate between 2 and 4 kΩ across clinical visits [9]. Monitoring electrode impedance fluctuations holds significant clinical value. By analyzing the extent of these fluctuations, clinicians can optimize electrical stimulation parameters, such as pulse duration. The purpose of this optimization is to establish “headroom” that separates comfortable loudness levels (C-levels) from electrode compliance limits. This headroom ensures that even during impedance increases, the delivered biphasic pulses remain unclipped, thereby maintaining an appropriate level of electrical stimulation.

Future research will investigate how targeted electrical stimulation modifications can mitigate or prevent rising impedance. This includes exploring the influence of the pulse duration, stimulation rate, and biphasic pulse polarity on electrode impedance in patients with cochlear implants. Interestingly, some electrodes show temporary impedance increases with stimulation, while their neighbors remain stable. This pattern can reverse on subsequent days, suggesting that impedance fluctuations are caused by local electrochemical reactions closer to the electrode surface offering varying levels of resistance to the flow of current to the cochlear electrolyte.

## 5. Conclusions

The analysis of electrode impedance subcomponents illuminated the impedance subcomponents contributing to rising or fluctuating electrode impedances. The observed decrease in impedance during device rest provides a unique opportunity to repeatedly measure and analyze subcomponent changes, identifying which components contribute to these impedance variations. This subcomponent analysis suggests that localized changes at the electrode interface, likely related to electrical stimulation and subsequent alterations in the electrochemical reactions at the electrode–electrolyte interface, are responsible for the observed impedance changes.

## Figures and Tables

**Table 1:** Subject demographics, implant type, duration of implant use, electrodes disabled, and clinical visit history for the 10 cochlear implant patients with fluctuating or rising electrode impedances.

Subject Number	Age	Gender	Implant Type	Duration of Implant Use	Electrodes Disabled	Clinical Visits (Days from Cochlear Implantation)
CI1	64	M	CI622	3 years	1, 2 (open)	173, 220, 1050
CI2	69	F	CI622	3 years	6 (open)	104, 113, 134, 167
CI3	68	M	CI522	6 years	None	856, 1675
CI4	68	F	CI522	6 years	None	673, 967, 974, 980
CI5	60	F	CI622	3 years	1, 2, 3	197, 201, 691
CI6	86	F	CI522	6 years	None	951, 1602
CI7	70	M	CI422	9 years	1, 2	1904, 2042
CI8	93	F	CI622	3 years	None	798, 1795
CI9	69	M	CI522	5 years	None	1070, 1071
CI10	71	M	CI622	2 years	None	164, 167

## Data Availability

The original contributions presented in this study are included in the article. Further inquiries can be directed to the corresponding author.
